# Effectiveness and Safety of Direct Oral Anticoagulants Versus Warfarin in Patients With Mechanical Heart Valves: A Systematic Review

**DOI:** 10.7759/cureus.89736

**Published:** 2025-08-10

**Authors:** Bethel T Mandefro, Sri Vidya Sundara, Xinyu Lu, Hamide Busmail, Sasika Weerakoon, Sravanthi Avula, Laraib Iftikhar

**Affiliations:** 1 Medicine, California Institute of Behavioral Neurosciences & Psychology, Fairfield, USA

**Keywords:** coagulation inhibitors, direct oral anticoagulant (doac), mechanical heart valve (mhv), prosthetic heart valve, warfarin

## Abstract

Patients with mechanical heart valves require lifelong anticoagulation primarily due to the thrombogenic nature of these valves. Warfarin has been the mainstay of therapy, but its drug-drug interactions and frequent monitoring requirements have diverted attention toward DOACs (direct oral anticoagulants) such as apixaban, rivaroxaban, and dabigatran. However, the use of DOACs in this group of patients remains controversial, particularly after negative outcomes in earlier trials. This systematic review focuses on the efficacy of DOACs compared to warfarin for preventing thromboembolic events and associated bleeding risks in patients with mechanical heart valves. Most evidence comes from observational studies and meta-analyses. More limited data exist for patients with mechanical heart valves. We systematically searched PubMed, PubMed Central, and Google Scholar for English-language studies published between January 2015 and May 5, 2025. Eligible studies included randomized controlled trials (RCTs), observational studies, systematic reviews, and meta-analyses comparing DOACs and warfarin in adults with mechanical heart valves and related comorbidities such as atrial fibrillation and valvular heart disease. Articles were screened and assessed using standard quality appraisal tools. We included three observational studies, two RCTs, and six systematic reviews and meta-analyses. Most of the studies favored warfarin as the standard therapy for patients with mechanical heart valves, while DOACs showed relatively inferior outcomes. Current practice guidelines from the American College of Cardiology Foundation/American Heart Association Task Force suggest warfarin as the standard treatment for patients with mechanical heart valves. There is a further need for long-term, high-quality clinical trials using a newer generation of DOACs among this high-risk group.

## Introduction and background

Mechanical valve replacement (MVR) is a critical intervention for patients with severe valvular heart disease (VHD), necessitating lifelong anticoagulation to prevent thromboembolic events associated with mechanical prostheses [1]. According to iData Research, approximately 100,000 mechanical and bioprosthetic valve replacement surgeries are performed annually in the United States. This number is projected to rise to 290,000 by 2029 [2]. Mechanical valves are generally favored over bioprosthetic options due to their long-term durability, particularly in younger patients [3]. However, they carry a higher risk of thrombosis and require lifelong anticoagulation therapy, which increases bleeding risk [3].

In clinical practice, dissatisfaction with frequent INR monitoring has increased interest in alternative anticoagulant options. Patients undergoing valve replacement often face the decision between a mechanical valve, which requires lifelong anticoagulation, and a bioprosthetic valve, which may deteriorate over time and necessitate reoperation [4].

Anticoagulation therapy has evolved over the years, initially with the introduction of unfractionated heparin, followed by warfarin, low-molecular-weight heparins (LMWH), and more recently, direct oral anticoagulants (DOACs). DOACs include dabigatran (a thrombin inhibitor), rivaroxaban, apixaban, and edoxaban (factor Xa inhibitors) [5].

Despite the emergence of DOACs, current guidelines from the American College of Cardiology Foundation/American Heart Association (ACCF/AHA) and the European Society of Cardiology (ESC) continue to recommend vitamin K antagonists (VKAs) as the standard anticoagulation therapy for patients with mechanical heart valves [6]. The RE-ALIGN trial, which compared dabigatran to warfarin in patients with mechanical heart valves, was terminated due to an increased incidence of both bleeding and thrombotic events in the DOAC group, indicating a higher risk without benefit [7].

Warfarin is relatively inexpensive but requires frequent follow-up and has more drug and food interactions. DOACs do not require regular monitoring and have a short half-life. Some also have reversal agents, which enhance their safety profile, such as idarucizumab for dabigatran and andexanet alfa for apixaban and rivaroxaban, representing a potential alternative to warfarin in selected populations [8,9].

In this systematic review, we aim to evaluate current evidence regarding the effectiveness of DOACs compared to warfarin in preventing thrombosis and the respective bleeding risks.

## Review

Method

This systematic review was conducted in accordance with the PRISMA 2020 (Preferred Reporting Items for Systematic Reviews and Meta-Analyses) guidelines.

Data source and search strategy

We searched PubMed, PubMed Central, and Google Scholar for relevant articles published up to May 5, 2025. Both regular keywords (e.g., mechanical heart valve, prosthetic heart valve, warfarin, direct oral anticoagulant, coagulation inhibitors) and Medical Subject Headings (MeSH) terms were used. Table 1 shows the details of the search strategy and results.

**Table 1:** A detailed Boolean search string.

Databases	Search strategy and keywords	Filters used	Search results
PubMed MeSH search	prosthetic heart valve OR mechanical heart valve OR valvular prosthesis OR tissue heart valve OR replacement heart valve OR artificial heart valve OR prosthetic heart valve OR mechanical heart prosthesis OR metallic heart valve OR bioprosthetic heart valve OR cardiac heart valve OR implantable heart valve OR ( "Heart Valve Prosthesis/adverse effects"(Majr) OR "Heart Valve Prosthesis/standards"(Majr) )) OR ( "Heart Valve Prosthesis/adverse effects"(Mesh:NoExp) OR "Heart Valve Prosthesis/standards"(Mesh:NoExp)) AND warfarin OR warfarin OR heparin OR dabigatran OR rivaroxaban OR apixaban OR edoxaban OR ( "Warfarin/adverse effects"(Majr) OR "Warfarin/blood"(Majr) OR "Warfarin/standards"(Majr) OR "Warfarin/therapeutic use" (Majr) OR "Warfarin/toxicity"(Majr) )) OR ("Warfarin/adverse effects")Mesh:NoExp)OR "Warfarin/blood"(Mesh:NoExp) OR "Warfarin/standards"(Mesh:NoExp)OR"Warfarin/therapeutic use"(Mesh:NoExp)OR "Warfarin/toxicity"(Mesh:NoExp) ) AND direct oral anticoagulant OR blood thinner OR anticoagulant OR antithrombotic OR antiplatelet OR thrombolytics OR coagulation inhibitors OR vitamin K antagonist OR direct oral anticoagulants OR heparinoids OR (( "Factor Xa Inhibitors/adverse effects"(Majr) OR "Factor Xa Inhibitors/blood"(Majr) OR "Factor Xa Inhibitors/standards"(Majr) OR "Factor Xa Inhibitors/therapeutic use"(Majr)OR "Factor Xa Inhibitors/toxicity"(Majr) )) OR ( "Factor Xa Inhibitors/adverse effects"(Mesh:NoExp) OR "Factor Xa Inhibitors/blood"(Mesh:NoExp) OR "Factor Xa Inhibitors/standards"(Mesh:NoExp) OR "Factor Xa Inhibitors/therapeutic use"(Mesh:NoExp)OR "Factor Xa Inhibitors/toxicity"(Mesh:NoExp))	10 years, Free full text, English, Human, (female and male) Adult (19 years)	377
PubMed advanced search	Warfarin, prosthetic heart valve, mechanical heart valve, direct oral anticoagulant, coagulation inhibitors	10 years, Free full text, English, Human, (female and male) Adult (19 years)	123
PubMed Central	Warfarin, prosthetic heart valve, mechanical heart valve, direct oral anticoagulant, coagulation inhibitors	10 years, Free full text, English, Human, (female and male) Adult (19 years)	321
Google Scholar	Warfarin, prosthetic heart valve, mechanical heart valve, direct oral anticoagulant, coagulation inhibitors	10 years, Free full text, English, Human, (female and male) Adult (19 years) Most prevalent articles.	47

We applied the inclusion and exclusion criteria. The retrieved articles were imported into EndNote, where duplicates were removed. Two independent reviewers screened the titles and abstracts for relevance to the study objective. Disagreements were resolved through discussion with a third reviewer.

Inclusion criteria

We included studies involving adults (≥18 years), published in English, available in full text, peer-reviewed, and published within the last 10 years. Eligible study designs included randomized controlled trials (RCTs), cohort studies, systematic reviews, and meta-analyses. We included studies that compared warfarin with DOACs in the context of mechanical heart valves or VHD, including patients with comorbid atrial fibrillation (AF). Studies were required to be indexed in reputable databases.

Exclusion criteria

Exclusion criteria included animal studies, editorials, opinion pieces, ongoing/unpublished research, and articles that were not available in full text. 

Quality appraisal and data extraction

To assess the quality of the included studies, we used established assessment tools. For observational studies, we used the Newcastle-Ottawa Scale; for RCTs, the Cochrane Risk of Bias tool; and for systematic reviews and meta-analyses, the AMSTAR checklist (assessment of multiple systematic reviews). Each article was screened using the appropriate quality appraisal tool by two individuals, and disagreements were resolved by a third person. Table 2 shows the quality appraisal of the included cohort studies based on the Newcastle-Ottawa Scale. Table 3 shows the quality appraisal of the included randomized controlled trials based on the Cochrane Risk of Bias tool. Table 4 shows the quality appraisal of the included systematic reviews and meta-analyses based on the AMSTAR checklist.

**Table 2:** Quality assessment of the included cohort studies.

Study	Representativeness of the exposed cohort	Selection of the non-exposed cohort	Assortment of exposure	Outcome not present at the start	Comparability	Assortment of outcomes	Adequacy of follow-up	Length of follow-up	Risk of bias
Kalra et al. (2021) [10]	*	*	*	*	**	*	*	*	9\9 low risk
Johannsson et al. (2024) [11]	*	*	*	*	**	*	*	*	9\9 low risk
Dawwas et al. (2024) [12]	*	*	*	*	**	*	*	*	9\9 low risk

**Table 3:** Quality appraisal of the included randomized controlled trials.

Study	Domain 1	Domain 2	Domain 3	Domain 4	Domain 5	Overall risk
Wang et al. (2024) [13]	Low risk	Some concern	Low risk	Low risk	Low risk	Low risk of bias
Jawitz et al. (2020) [14]	Low risk	Some concern	Low risk	Low risk	Low risk	Low risk of bias

**Table 4:** Quality assessment of the included systematic reviews and meta-analyses.

Amstar domain	Srivastava et al. (2024) [15]	de Souza Lima Bitar et al. (2019) [16]	He et al. (2019) [17]	Uimonen et al. (2024) [18]	Pan et al. (2017) [19]	Kido et al. (2024) [20]
PICO component clearly stated?	Yes	Yes	Yes	Yes	Yes	Yes
Protocol registered before review started?	No	No	No	No	Yes	No
Explanation of the study design inclusion?	Yes	Yes	Yes	Yes	Yes	Yes
Comprehensive literature search performed?	Yes	Yes	Yes	Yes	Yes	Yes
Duplicate study selection?	Yes	Yes	Yes	Yes	Yes	Yes
Duplicate data extraction?	Yes	Yes	Yes	Yes	Yes	Yes
List of excluded studies with justification?	No	No	No	No	No	No
Detailed description of included studies?	Yes	Yes	Yes	Yes	Yes	Yes
Risk of bias assessment of included studies?	Yes	Yes	Yes	Yes	Yes	Yes
Funding sources of included studies reported?	No	No	No	No	No	No
Appropriate meta-analysis Method used?	Yes	Yes	Yes	Yes	Yes	Yes
Risk of bias considered in interpreting results?	Unclear	Unclear	Yes	Unclear	Yes	Unclear
Explanation of heterogeneity?	Yes	Yes	Yes	Yes	Yes	Yes
Publication bias assessment?	Yes	Yes	Yes	Yes	Yes	Yes
Funding and conflict of interest for the review?	No	Unclear	No	Yes	Yes	Yes
Overall	High	High	High	High	High	High

**Figure 1:**
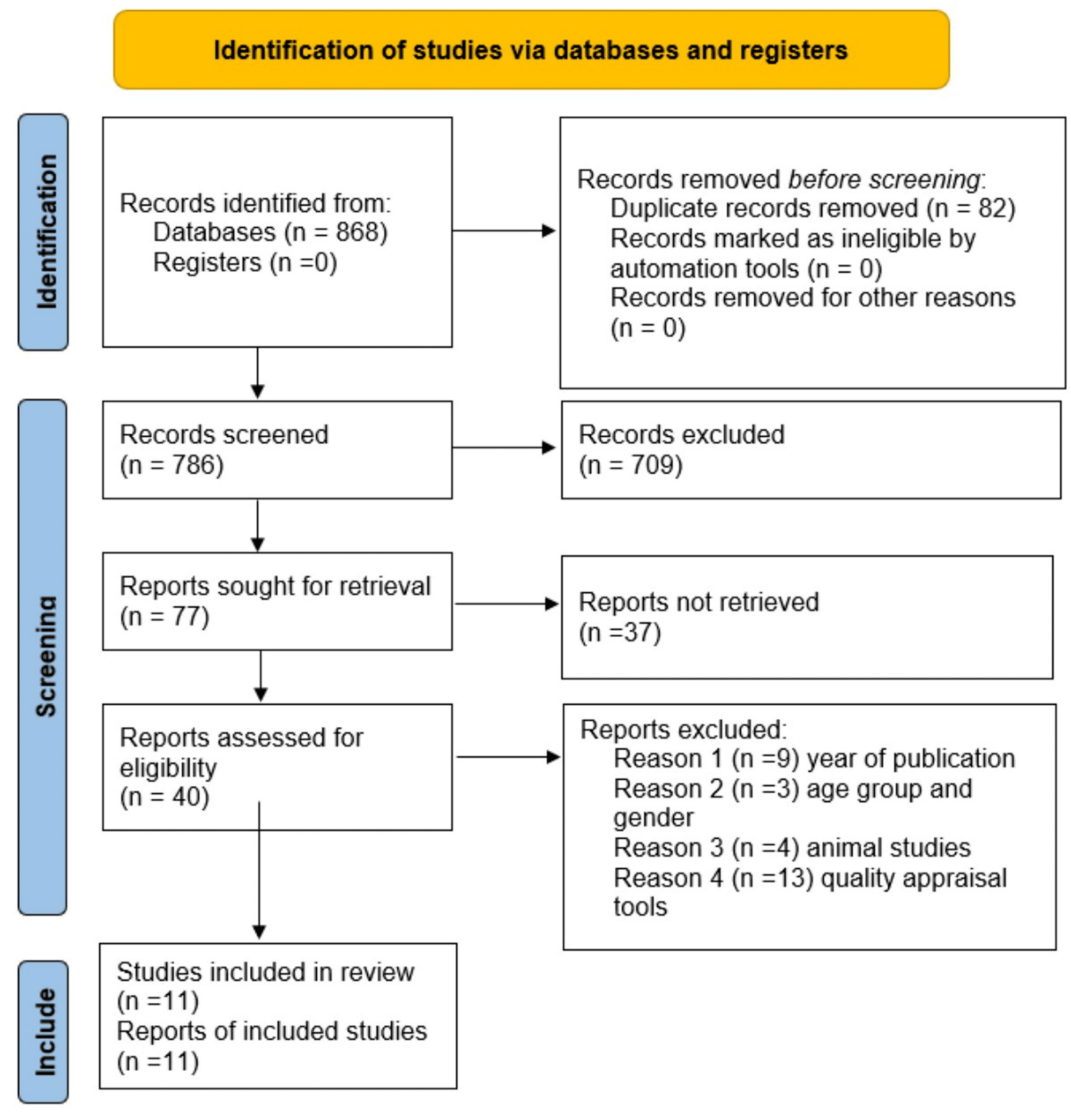
Selection process and the number of articles in a PRISMA flow chart PRISMA: Preferred Reporting Items for Systematic Reviews and Meta-Analyses.

Data were extracted from the remaining articles. For the observational studies and RCTs, we collected the author’s name, type of study, year of publication, country where the study was conducted, number of patients, duration, type of anticoagulants used, and outcomes. For systematic reviews and meta-analyses, we extracted the authors’ names, year of publication, study type, anticoagulants used, number of articles included, PRISMA compliance, and main outcomes (Figure 1).

Result

A total of 868 articles were identified from the database search. From these articles, 82 duplicates were removed. The remaining articles were screened based on the titles, which excluded 709 articles that were irrelevant to our topic. After reviewing the abstracts, 23 articles were removed due to the unavailability of full text. From the remaining articles, 14 that did not meet the eligibility criteria were removed. Based on animal studies, year of publication, age, and not meeting the target population, 16 articles were removed. A total of 24 articles were screened for quality appraisal, and 13 were removed for not fulfilling the quality appraisal requirements. The remaining 11 articles were included in this systematic review.

Of the 11 studies, six were systematic reviews and meta-analyses, three were observational studies, and two were randomized controlled trials. The two randomized controlled trials were followed for two years. The observational studies were conducted for three, seven, and nine years. Two of the observational studies and both randomized controlled trials were conducted in North America (Table 5).

**Table 5:** The characteristics of the observational studies and RCTs. DOAC: direct oral anticoagulant, VTE: venous thromboembolism, DVT: deep vein thrombosis, PTE: pulmonary thromboembolism, RCT: randomized controlled trial, TE: thromboembolic event.

Author name	Type of study	Year of publication	Country	Number of patients	Duration of the study	Type of anticoagulant	Outcome
Study 1: Kalra et al. [10]	Cohort	March 2021	USA	177,915	2014-2017	Compared both warfarin and DOACs	Higher rates of bleeding, VTE, DVT, and PTE in groups that received DOACs
Study 2: Johannsson et al. [11]	Retrospective observational	December 2023	Canada	671	2010-2019	Warfarin in a mechanical heart valve	Those with poor control had a thromboembolic event
Study 3: Dawwas et al. [12]	Cohort	August 2024	USA	81,667	2014-2021	Compared warfarin with DOACs on valvular heart disease	DOACs were associated with a lower risk of ischemic stroke, systemic embolism, and bleeding compared to warfarin
Study 4: Wang et al. [13]	RCT	May 2023	USA	863	2020-2022	Compared apixaban and warfarin	Higher rate of TE in the apixaban group
Study 5: Jawitz et al. [14]	RCT	September 2020	USA	1,000	2 Years	Compared apixaban and warfarin on a mechanical heart valve	Discontinued because of significant thrombosis risk in the apixaban group

Two of the RCTs were conducted over two years, involving 1,863 patients in the United States, and showed that the use of apixaban compared to warfarin was associated with an increased risk of thromboembolism. Two cohort studies involving 178,586 patients reported higher bleeding rates, higher venous thromboembolism, deep vein thrombosis, and pulmonary embolism in groups that received DOACs. Thromboembolic events were observed in those with poor control of warfarin use. One cohort study involving 81,667 patients comparing DOACs with warfarin in VHD showed a lower risk of thromboembolism, systemic embolism, and stroke compared to warfarin (Table 6).

**Table 6:** The characteristics of the systematic review that are included in the review. DOAC: direct oral anticoagulant, AF: atrial fibrillation, VHD: valvular heart disease.

Study ID	Reference	Year of publication	Review type	Anticoagulant compared	Number of studies included	PRISMA compliance	Main outcome
6	Srivastava et al. [15]	2024	Systematic review and meta-analysis	Warfarin versus DOACs	8	Yes	Warfarin is preferred over DOACs
7	de Souza Lima Bitar et al. [16]	2019	Systematic review and meta-analysis	Warfarin versus DOACs	6	Yes	DOACs have significantly reduced the risk of stroke and intracranial bleeding compared to warfarin in AF and VHD, but not mechanical heart valves
8	He et al. [17]	2019	Meta analysis	Warfarin versus DOACs	6	Yes	DOACs show reduced risk of stroke and systemic thromboembolism in VHD but not major bleeding than warfarin
9	Uimonen et al. [18]	2024	Meta analysis	All anticoagulant	8	Yes	Potential advantage of anticoagulation
10	Pan et al. [19]	2017	Systematic review and meta-analysis	Warfarin and DOACs	4	Yes	DOACs, as compared to warfarin, have reduced risk in stroke, systemic embolism, and intracranial hemorrhage in patients with AF and with or without VHD
11	Kido et al. [20]	2021	Meta analysis	Warfarin and DOACs	8	Yes	No difference in stroke and systemic embolism, and with warfarin and DOACs, but decreased risk of bleeding with DOACs as compared to warfarin

Discussion

This systematic review focused on the current evidence regarding the effectiveness of DOACs compared to warfarin in patients with mechanical heart valves, particularly in terms of preventing thrombosis and reducing the risk of bleeding associated with the two drugs. From the studies collected, we found that DOACs were less effective than warfarin in these patients.

Safety and efficacy of DOACs and warfarin

Warfarin remains a superior option for preventing thromboembolic complications in patients with mechanical heart valves. Most of the included studies in our review found that DOACs performed less effectively in the prevention of thromboembolic complications compared to warfarin in this population. For instance, a study by Srivastava et al. [15] reviewed eight articles on both mechanical and bioprosthetic valves and reported that patients on DOACs had a thrombosis rate of 5.8% compared to 1.8% in the warfarin group, concluding that warfarin was a safer option, while DOACs were associated with higher thrombosis rates. In a similar study by Wang et al. [13] comparing warfarin with apixaban on X mechanical aortic valves, the apixaban group experienced a thromboembolic event rate of 3.9% per patient-year compared to 1.5% per patient-year in the warfarin group, indicating a significantly higher risk of thromboembolic events with apixaban.

In line with these findings, Kalra et al. [10] analyzed data from a large cohort of 177,915 patients with both mechanical and bioprosthetic valves and observed a higher risk of bleeding, venous thromboembolism (VTE), venous thrombosis (VT), and pulmonary thromboembolism (PTE) in the groups that received DOACs. Kalra et al. [10] also noted that despite the risks documented in prior analyses, DOACs were used off-label in patients with mechanical heart valves, especially in those with bioprosthetic valves and AF. Underdosing was common in elderly patients and those with renal impairment, and it was associated with a higher rate of thromboembolic events. Overdosing was linked to an increased risk of bleeding complications, including gastrointestinal and intracranial bleeding. However, the overall trend was unfavorable to choosing DOACs over warfarin in patients with mechanical heart valves.

Additionally, due to safety concerns and an increased risk of complications, some trials investigating DOACs were terminated early. For instance, the study by Jawitz et al. [14], which enrolled 1,000 patients with on X mechanical valves over two years, was discontinued after a significantly higher incidence of thrombosis was observed in the apixaban group.

To sum up, many studies favor the use of warfarin over DOACs for patients with mechanical heart valves. Future research should focus on more randomized controlled trials to assess the safety and efficacy of DOACs; until then, warfarin should remain the primary medication.

Use of DOACs compared to warfarin in VHD and AF

While the use of DOACs was shown to be less favorable in patients with mechanical heart valves compared to warfarin, they showed variable results in patients with VHD and AF. For example, a meta-analysis by Kido et al. [20], which reviewed eight studies on the management of AF in patients with VHD, found that DOACs failed to show additional benefits in reducing major bleeding and all-cause mortality but did decrease the risk of stroke and systemic embolism compared to warfarin.

Studies support the safety and efficacy of DOACs in patients with non-mechanical VHD and AF. Similarly, a large observational study by Dawwas et al. [12] involving 81,667 patients concluded that DOACs were associated with a lower risk of ischemic stroke, systemic embolism, and bleeding compared to warfarin in VHD patients.

Additional supporting evidence comes from the study by de Souza Lima Bitar et al. [16], which reported that DOACs were a safer option for preventing intracranial bleeding, thrombosis, and stroke in patients with AF and VHD, but not in those with mechanical heart valves.

In a similar study, DOACs appeared to offer superior outcomes compared to warfarin in preventing thromboembolic events without an increased risk of major bleeding. He et al. [17] found that DOACs were associated with a reduced risk of stroke and systemic thromboembolism in VHD, but not major bleeding, compared to warfarin; they had a comparable effect to warfarin. In addition, patients on DOACs had fewer bleeding events compared to those on warfarin.

These studies suggest that DOACs have a role in preventing thrombosis and reducing the risk of bleeding in selected patients, particularly those with AF and VHD without mechanical heart valves. Further studies are required to confirm their effectiveness in borderline patients and those with additional comorbidities.

In conclusion, DOACs may offer an effective alternative to warfarin in patients with VHD and AF. Additional studies are needed to strengthen the evidence base.

Benefits of using anticoagulants

The importance of anticoagulation in patients with mechanical heart valves is well established, particularly for preventing thromboembolic complications. Some of the studies we reviewed showed similar evidence. In a study by Johannsson et al. [11] involving 671 patients with mechanical valves, the use of warfarin in patients with mechanical heart valves demonstrated that those who did not have adequate anticoagulation control experienced thromboembolic events. This suggests that good INR control, strict follow-up, and dose adjustment as needed are essential with anticoagulants such as warfarin.

In contrast, for patients with AF and non-mechanical VHD, DOACs may offer a better balance between safety and efficacy. In a study by Pan et al. [19] involving patients with native and bioprosthetic valves, DOACs compared to warfarin significantly reduced the risk of stroke, systemic embolism, and intracranial hemorrhage in patients with AF and with or without VHD. This finding supports the potential for DOACs to be appropriate for non-mechanical valve-related indications, especially when anticoagulation monitoring poses challenges, such as in patients with poor INR control or limited access to regular laboratory testing. However, further studies are still required.

Limitations

A key limitation of this review is the limited number of randomized clinical trials, many of which included small sample sizes. Additionally, most observational studies had short follow-up durations, restricting the ability to assess long-term outcomes. Some potentially relevant articles could not be included due to lack of access to full-text versions. Several of the included studies did not specify the generation or type of valve used, limiting our ability to assess the impact of valve design on outcomes. Furthermore, many studies grouped patients with AF and VHD together without clearly differentiating subgroups, such as mechanical versus bioprosthetic valves, or distinguishing between native valve disease and post-valve replacement. To address this, we attempted to analyze and discuss these subgroups separately within the discussion section.

## Conclusions

This systematic review evaluated the comparative use of DOACs and warfarin in patients with mechanical heart valves. The review consistently found that the use of DOACs is limited and raises concerns about thromboembolic events. Current evidence favors warfarin, despite the requirement for frequent INR monitoring. DOACs require dose adjustment in certain situations, such as impaired renal function, advanced age, or concurrent use with other medications. Their use may not be recommended for mechanical heart valves because the risks outweigh the benefits. However, DOACs have demonstrated promising efficacy in patients with AF and VHD that does not involve mechanical valves. Given the thrombogenic nature of most mechanical heart valves and the high risk to patients if not on proper anticoagulation, current guidelines recommend warfarin as the first-line anticoagulant.

Currently available clinical trials have been conducted on a limited number of patients over a short period. We recommend the need for high-quality, large-population, longer-duration clinical trials with newer formulations of DOACs, such as rivaroxaban. Future research should prioritize evaluating outcomes in patients with various comorbidities, valve types, genetic profiles, and long-term drug use. These conclusions should be interpreted with caution, as most of the included studies were observational and varied in patient populations, valve types, and study designs. Meanwhile, warfarin remains the gold standard treatment for the prevention of thromboembolic events in patients with mechanical heart valves.
